# Supplementary material for: Heat adaptation of phage T7 under an extended genetic code
Source: Virus Evol. 2021 Dec 1;7(2):veab100. doi: 10.1093/ve/veab100 (PMC8923235; doi:10.1093/ve/veab100)
Supplement: veab100_Supp [file veab100_supp.zip › Sequencing_Analysis.zip!/Sequencing_Analysis/A1/evidence/JC_55.html]

BRESEQ :: Evidence


| New junction evidence | | | | | | | | | | | |
| --- | --- | --- | --- | --- | --- | --- | --- | --- | --- | --- | --- |
|  | | seq id | position | reads (cov) | reads (cov) | score | skew | freq | annotation | gene | product |
| \* | ? | NC\_001604.1 | = 13244 | 2141 (0.690) | 49 (0.020) | 39/582 | 27.3 | 4.5% | intergenic (–/–) | *–/–* | –/– |
|  | ? | NC\_001604.1 | 14067 = | 6 (0.000) |  |  |  |  | intergenic (–/–) | *–/–* | –/– |
| Rejected: Coverage evenness skew score above cutoff. | | | | | | | | | | | |
| Rejected: Frequency below/above cutoff threshold. | | | | | | | | | | | |

Only 100 of 122 total aligned reads displayed.  

`TGGTGCTGGTCGTAATTTGTCACCTTAAGAACCCAGACAAAGGTAAAGCACATGAGGAAGGTCGCCCCGTTTCTATTACTGACCTACGTGGTTCTGGCGCACTACGCCAACTATCTGATACTATTATTGCCCTTGAGCGTAATCAGCAAGGCGATATGCCTAACCTTGTCCTCGTTCGTATTCTCAAGTGCCGCTTTACTGGTGATACTGGTATCGCTGGCTACATGGAATACAACAAGGAAACCGGATGGCTTGAACCATCAAGTTACTCAGGGGAAGAAGAGTCACACTCAGAGTCAACAGACTGG‑‑‑‑‑‑‑‑‑‑‑‑‑‑‑‑‑‑‑‑‑‑‑‑‑‑‑‑‑‑‑‑‑‑‑‑‑‑‑‑‑‑‑‑‑‑‑‑‑‑‑‑‑‑‑‑‑‑‑‑‑‑‑‑‑‑‑‑‑‑‑‑‑‑‑‑‑‑‑‑‑‑‑‑‑‑‑‑‑‑‑‑‑‑‑‑‑‑‑‑‑‑‑‑‑‑‑‑‑‑‑‑‑‑‑‑‑‑‑‑‑‑‑‑‑‑‑‑‑‑‑‑‑‑‑‑‑‑‑‑‑‑‑‑‑‑‑‑‑‑‑‑‑‑‑‑‑‑‑‑‑‑‑‑‑‑‑‑‑‑‑‑‑‑‑‑‑‑‑‑‑‑‑‑‑‑‑‑‑‑‑‑‑‑‑‑‑‑‑‑‑‑‑‑‑‑‑‑‑‑‑‑‑‑‑‑‑‑‑‑‑‑‑‑‑‑‑‑‑‑‑‑‑‑‑‑‑‑‑‑‑‑‑‑‑‑‑‑‑‑‑‑‑‑‑‑‑‑‑‑‑‑‑‑‑‑‑‑‑‑‑‑‑‑‑‑‑‑‑‑‑‑‑‑‑‑‑‑‑‑  >  NC_001604.1/12937‑13244`  
`‑‑‑‑‑‑‑‑‑‑‑‑‑‑‑‑‑‑‑‑‑‑‑‑‑‑‑‑‑‑‑‑‑‑‑‑‑‑‑‑‑‑‑‑‑‑‑‑‑‑‑‑‑‑‑‑‑‑‑‑‑‑‑‑‑‑‑‑‑‑‑‑‑‑‑‑‑‑‑‑‑‑‑‑‑‑‑‑‑‑‑‑‑‑‑‑‑‑‑‑‑‑‑‑‑‑‑‑‑‑‑‑‑‑‑‑‑‑‑‑‑‑‑‑‑‑‑‑‑‑‑‑‑‑‑‑‑‑‑‑‑‑‑‑‑‑‑‑‑‑‑‑‑‑‑‑‑‑‑‑‑‑‑‑‑‑‑‑‑‑‑‑‑‑‑‑‑‑‑‑‑‑‑‑‑‑‑‑‑‑‑‑‑‑‑‑‑‑‑‑‑‑‑‑‑‑‑‑‑‑‑‑‑‑‑‑‑‑‑‑‑‑‑‑‑‑‑‑‑‑‑‑‑‑‑‑‑‑‑‑‑‑‑‑‑‑‑‑‑‑‑‑‑‑‑‑‑‑‑‑‑‑‑‑‑‑‑‑‑‑‑‑‑‑‑‑‑‑‑‑‑‑‑‑‑‑‑‑‑‑‑‑‑‑‑‑‑‑‑acagactggCTGGAAGAAACCAGAAGTTACCAAGCTGAGTCATAAGGTGTTCGATAAGGACACTATGACCCACATCAAGGCTGGTGATTGGGTTAAGGTTGACATGGGAGTTGTTGGTGGATACGGCTACGTCCGCTCAGTTAGTGGCAAATATGCACAAGTGTCATACATCACAGGTGTTACTCCACGCGGTGCAATCGTTGCCGATAAGACCAACATGATTCACACAGGTTTCTTGACAGTTGTTTCATATGAAGAGATTGTTAAGTCACGATAATCAATAGGAGAAATCAATATGA  >  NC_001604.1/14067‑14356`  

                                                                                                                                                                                                                                                                                                                                                                                                                                                                                                                                                                                                                        
`gggctcggagatgtggtataagagacagGAACCCAGACAAAGGTAAAGCACATGAGGAAGGTCGCCCCGTTTCTATTACTGACCTACGTGGTGCTGGCGCACTACGCCAACTATCTGATACTATTATTGCCCTTGAGCGTAATCAGCAAGGCGATATGCCTAACCTTGTCCTCGTTCGTATTCTCAAGTGCCGCTTTACTGGTGATACTGGTATCGCTGGCTACATGGAATACAACAAGGAAACCGGATGGCTTGAACCATCAAGTTACTCAGGGGAAGAAGAGTCACACTCAGAGTCAAC                                                                                                                                                                                                                                                                                                           <  1:82715/273‑1`  
`gGGTGCTGGTCGTAATTTGTCACCTTAAGAACCCAGACAAAGGTAAAGCACATGAGGAAGGTCGCCCCGTTTCTATTACTGACCTACGTGGTTCTGGCGCACTACGCCAACTATCTGATACTATTATTGCCCTTGAGCGTAATCAGCAAGGCGATATGCCTAACCTTGTCCTCGTTCGTATTCTCAAGTGCCGCTTTACTGGTGATACTGGTATCGCTGGCTACATGGAATACAACAAGGAAACCGGATGGCTTGAACCATCAAGTTACTCAGGGGAAGAAGAGTCACACTAAGAGTCAAC                                                                                                                                                                                                                                                                                                           <  1:477584/300‑1`  
`gGGTGCTGGTCGTAATTTGTCACCTTAAGAACCCAGACAAAGGTAAAGCACATGAGGAAGGTCGCCCCGTTTCTATTACTGACCTACGTGGTTCTGGCGCACTACGCCAACTATCTGATACTATTATTGCCCGTGAGCGTAATCAGCAAGGCGATATGCCTAACCTTGTCCTCGTTCGTATTCTCAAGTGCCGCTTTACTGGTGATACTGGTATCGCTGGCTACATGGAATACAACAAGGAAACCGGATGGCTTGAACCATCAAGTTACTCAGGGGAAGAAGAGTCACACTCAGAGTCAAC                                                                                                                                                                                                                                                                                                           <  1:11951/300‑1`  
`cggagatgtgtataagagacagCCTTAAGAACCCAGACAAAGGTAAAGCACATGAGGAAGGTCGCCCCGTTTCTATTACTGACCTACGTGGTTCTGGCGCACTACGCCAACTATCTGATACTATTATTGCCCTTGAGCGTAATCAGCAAGGCGATATGCCTAATCTTGTCCTCGTTCGTATTCTCAAGTGCCGCTTTACTGGTGATACTGGTATCGCTGGCTACATGGGATACAACAAGGAAACCGGATGGCTTGAACCATCAAGTTACTCAGGGGAAGAAGAGTCACACTCAGAGCCAAC                                                                                                                                                                                                                                                                                                           <  1:242270/279‑1`  
`agagacaGGTAGTAATTTGTCACCTTAAGAACCCAGACAAATGTAAAGCACATGAGGAAGGTCGCCCCGTTTCTATTACTGACCTACGTGGTTCTGGCGCACTACGCCAACTATCTGATACTATTATTGCCCGTGAGCGTAATCAGCAAGGCGATATGCCTAACCTTGTCCTCGTTCGTATTCTCAAGTGCCGCTTTACTGGTGATACTGGTATCGCTGGCTACATGGAATACAACAAGGAAACCGGATGGTTTGAACCATCAAGTTACTCAGGGGAAGAAGAGTCACACTCAGAGTCAAC                                                                                                                                                                                                                                                                                                           <  1:331723/294‑1`  
`TGGTGCTGGTCGTAATTTGTCACCTTAAGAACCCAGACAAAGGTAAAGCACATGATGAAGGTCGCCCCGTTTCTATTACTGACCTACGTGGTTCTGGCGCACTACGCCAACTATCTGATACTATTATTGCCCTGTAGCGTAATCAGCAAGGCGATATGCCTAACCTTGTCCTCGTTCGTATTCTCAAGTGCCGCTTTACTGGTGATACTGGTATCGCTGGCTACATTGAATACAACAAGGAAACCGGATGGCTTGAACCATCAAGTTACTCAGGGGAAGAAGAGTCACACTCAGAGTCAAC                                                                                                                                                                                                                                                                                                           <  1:414242/301‑1`  
`TGGTGCTGGTCGTAATTTGTCACCTTAAGAACCCAGACAAAGGTAAAGCACATGAGGAAGGTCGCCCCGTTTCTATTACTGACCTACGTGGTTCTGGCGCACTACGCCAACTATCTGATACTATTATTGCCCTTGAGCGTAATCAGCAAGGCGATATGCCTAACCTTGTCCTCGTTCGTATTCTCAAGTGCCGCTTTACTGGTGATACTGGTATCGCTGGCTACATGGAATACAACAAGGAAACCGGATGGCTTGAACCATCAAGTTACTCAGGGGAAGAAGAGTCACACTCAGAGTCAAC                                                                                                                                                                                                                                                                                                           <  1:132778/301‑1`  
 `GGTGTTGGTCGGATTTGTTCAACTTAAGAACCCAGACAAAGGTAAAGAACATGAGGTAGGTCGCCCCGTTTCGATTACGGAACTACGTGATTCTGGCGCATAAGGCCAACTATCGGATACTATTATTGCCCTCGAGCGTAAACAGCAAGGCGATATGCCGAACCTTGTCCTCGTTCGTATTCTCAAGTGCCGCTTGACTGGTGATACTGGTATCGCTGGCTACATGGAATACAACAAGGGAACCGGATGGCTTGAACCATCAAGTTACTCAGGGGAAGAAGAGTCACACTCAGAGTCAACA                                                                                                                                                                                                                                                                                                          <  1:214737/301‑1`  
 `GGTGCTGGTCGTAATTTGTCACCTTAAGAACCCAGACAAAGGTAAAGCACATGAGGAAGGTCGCCCCGTTTCTATTACTGACCTACGTGGTTCTGGCGCACTACGCCAACTATCTGATACTATTATTTCCCTTGAGCGTAATCAGCAAGGCGATATGCCTAACCTTGTCCTCGTTCGTATTCTCAAGTGCCGCTTTACTGGTGATACTGGTATCGCTGGCTACATGGAATCCAACAAGGCAACCGGATGGCTTGAACCATCAAGTTACTCAGGGGAAGAAGAGTCACACTCAGAGTCAACc                                                                                                                                                                                                                                                                                                          >  1:177442/1‑300`  
 `GGTGCTGGTCGTAATTTGTCACCTTAAGAACCCAGACAAAGGTAAAGCACATGAGGAAGGTCGCCCCGTTTCTATTACTGACCTACGTGGTTCTGGCGCACTACGCCAACTATCTGATACTATTATTGCCCTTGAGCGTAATCAGCAAGGCGATATGCCTAACCTTGTCCTCGTTCGTATTCTCAAGTGCCGCTTTACTGGTGATACTGGTATCGCTGGCTACATGGAATACAACAAGGAAACCGGATGGCTTGAACCATCAAGTTACTCAGGGGAAGAAGAGTCACACTCAGAGTAAACA                                                                                                                                                                                                                                                                                                          >  1:425998/1‑301`  
  `gcGCTCGTCGTAATTTGTCACCGTAAGCACCCAGACAAAGGTAAAGCACATGAGGAAGGTCGCCCCGTTTCTAGTACTGACCTACGTGGTTCTGGCGAACTACGCAAACTATCTGATACTATTATTGCCCTTGAGCGTAATCAGCAAGGGGATATGCCTAACCTTGTCCTCGTTCGTATTCTCAAGTGCCGCTTTACTGGTGATACGGGTATCGCTGGCTACATGGAATACAACAAGGAAACCAGATGGCTTGAACCATCAAGTTACTCAGGGGAAGAAGAGTCACACTCAGAGTCAACAG                                                                                                                                                                                                                                                                                                         <  1:340341/299‑1`  
  `GTGCTGGTCGTAATTTGTCATCTTAAGAACACAAACAAAGGTAAAGCACATTAGGAAGGTAGCTCCTGTTCTATTACTGAACTACGTGGTTCTGGCGCACTACGCCAACTATCGGATACTATTATTGCCCTTGAGCGTAATCAGCAAGGCGATATGCCTAACCTTGTCCTCGTTCGTATTCTCAAGTGCCGCTTTACTGGTGATACTGGTATCGCTGGCTACATGGAATACAACAAGGAAACCGGATGGCTTGAACCATCAAGTTACTCAGGGGAAGAAGAGTCACACTCAGAGTCAACAG                                                                                                                                                                                                                                                                                                         <  1:136455/301‑1`  
  `GTGCTGGTCGTAATTTGTCACCTTAAGAACCCAGACAAAGGTAAAGCACATGAGGAAGGTCGCCCCGTTTCTATTACTGACCTACGTGGTTCTGGCGCACTACGCCAACTATCTGATACTATTATTGCCCTTGAGCGTAATCAGCAAGGCGATATGCCTAACCTTGTCCTCGTTCGTATTCTCAATTGCCGCTTTACTGGTGATACTGGTATCGCTGGCTACATGGACTACAACAAGGAAACCGGATGGCTTGAACCATAAAGTTACTCAGGGGAAGAAGAGTAACACCACGAGTCAACAG                                                                                                                                                                                                                                                                                                         >  1:109294/1‑301`  
  `GTGCTGGTCGTAATTTGTCACCTTAAGAACCCAGACAAAGGTAAAGCACATGAGGAAGGTCGCCCCGTTTCTATTACTGACCTACGTGGTTCTGGCGCACTACGCCAACTATCTGATACTATTATTGCCCTTGAGCGTAATCAGCAAGGCGATATGCCTAACCTTGTCCTCGTTCGTATTCTCAAGTGCCGCTTTACTGGTGATACTGGTATCGCTGGCTACATGGAATACAACAAGGAAACCGGATGGCTTGAACCATCAAGTTACTCAGGGGAAGAAGAGTCACACTCAGAGTGAACAG                                                                                                                                                                                                                                                                                                         <  1:401914/301‑1`  
  `GTGCTGGTCGTAATTTGTCACCTTAAGAACCCAGACAAAGGTAAAGCACATGAGGAAGGTCGCCCCGTTTCTATTACTGACCTACGTGGTTCTGGCGCACTACGCCAACTATCTGATACTATTATTGCCCTTGAGCGTAATCAGCAAGGCGATATGCCTAACCTTGTCCTCGTTCGTATTCTCAAGTGCCGATTTACTGGTGATACTGGTATCGCTGGCTACATGGAATACAACAAGGAAACCGGATGGCTTGAACCATCAAGTTACTCAGGCGAAGAAGAGTCACACTCAGAGTCAACAG                                                                                                                                                                                                                                                                                                         >  1:168042/1‑301`  
  `GTGCTGGTCGTAATTTGTCACCTTAAGAACCCAGACAAAGGTAAAGCACATGAGGAAGGTCGCCCCGTTTCTATTACTGACCTACGTGGTTCTGGCGCACTACGCAAACTATCTGATACTATTATTGCCCTTGAGCGTAATCAGCAAGGCGATATGCCTAACCTTGTCCTCGTTCGTATTCTCAAGTGCCGCTTTACTGGTGATACTGGTATCGCTGGCTACATGGAATACAACAAGGAAACCGGATGGCTTGAACCATCAAGTTACTCAGGGGAAGAAGAGTCACACTAAGGGTCAACAa                                                                                                                                                                                                                                                                                                         >  1:208315/1‑300`  
  `GTGCTGGTCGTAATTTGTCACCTTAAGAACCCAGACAAAGGTAAAGCACATGACGAAGGTCGCCCCGTTTCTATTACTGACCTACGTGGTTCTGGCGCACTACGCCAACTATCTGATACTATTATTGCCCTTGAGCGTAATCAGCAAGGCGATATGCCTAACCTTATCCTCGTTCGTATTCTCAAGTGCCGCTTTACTGGTGATACTGGTATCGCTGGCTACATGGAATACAACAAGGAAACCGGATGGCTTGAACCATCAAGTTACTCAGGGGAAGAAGAGTCACACTCAGAGTCAACAG                                                                                                                                                                                                                                                                                                         <  1:27134/301‑1`  
  `GTGCTAGTCGTAATTTGTAACCTTACTAACCCAGGCAAAGGTAAAGAACATGACGAAGGTCGCCCCGTTTCTATTACTAACCTACGTGGTTCTGGCGAACTACGCCAACTATCTGATACTATTATTGCCCTTGAGCGTAATCAGCAAGGCGATATGCCTAACATTGTCCTCGTTCGTATTCTCAAGTGCCGCTTTACTGGTGATACTGGTATCGCTGGCTACATGGAATACAACAAGGAAACCGGATGGCTTGAACCATCAAGTTACTCAGGGGAAGAAGAGTCACACTCAGAGTCAACAG                                                                                                                                                                                                                                                                                                         <  1:485914/301‑1`  
   `TGCTGGTCGTAATTTGTCACC‑TAACAACCCAGACAAAGGTAAAGCACATGAGGAAGGTCGCCCCGTTTCTCTTACTGACCTACGTGGTTCTGGCGCACTACGCAAACTATCTGATACTATTATTGCCCTTGAGCGTAATCAGCAAGGCGATATGCCCAACCTTGTCCTCGTTCGTATTCTCAAGTACCGCTTTACTGGTGATACTGGTATCGCTGGCTACATGGAATACAACAAGGAAACCGGATGGCTTGAACCATCAAGTTACTCAGGGGAAGAAGAGTCACACTCAGAGTCAACAGAC                                                                                                                                                                                                                                                                                                       <  1:354101/301‑1`  
    `taTGGTCGTAATTTGTCAACTTAAGAACCCAGACAAAGGTAAAGCACATGAGGAAGGGCGACCCGTTTCTAGTACTGACCTACGGGGTTCTGGCGAACTACGCCAACTATCTGATACAATTATTGCCCTTGAGCGTAATCAGCAAGGCGATATGCCGAACCTTGTCCTCGTTCGTATTCTCAAGTGCCGCTTTACTGGTGATACTGGTATCGCTGGCTACATGGAATACAACAAGGAAACCGGATGGCTTGAACCATCAAGTTACTCAGGGGAAGAAGAGTCACACTCAGAGTCAACAGAC                                                                                                                                                                                                                                                                                                       <  1:337743/299‑1`  
    `gcgGGTCGTAATGTGTCACCTTAAGAACCCAGACAAAGGTAAAGCACATGAGGAAGGTCGCCCCGTTTCTATTACTGACCGACGTGTGTCTGGCGCACTACGCCAACTATCTGATACTATTATTGCCCTTGAGCGTAATCAGCAAGGAGATATGCCTAACCTTGTCCTCGTTCGTATTCGCAAGTGCCGCTTTACTGGTGATACTGGTATCGCTGGCTACATGGAATACAACAAGGAAACCGGATGGCTTGAACCAGCAAGTTACTCAGGGGAAGAAGAGTCACACTCAGAGTCAACAGAC                                                                                                                                                                                                                                                                                                       <  1:262518/298‑1`  
    `gatgtgtataagagacagCCTTAAGAACCCAGACAAAGGTAAAGCACATGAGGAAGGTCTCCCCGTTTCTATTACTGACCTACGTGGTTCTGGCGCACTACGCCAACTATCTGATACTATTATTGCCCTTGATCTTAATCAGCAAGGCGATATGCCTAACCTTGTCCTCGTTTGTATTCTCAAGTGCCGCTTTACTGGTGATACTGGTATCGCTGGCTACATGGAATACAACAAGGAAACCGGATGGCTTGAACCATCAAGTTACTCAGGGGAAGAAGAGTCACACTCAGAGTCAACAGAC                                                                                                                                                                                                                                                                                                       <  1:421882/283‑1`  
    `gatcggagatgtgtgtcagagacAGAACCTAGACAAAGGGAACGCACATGAGGAAGGTCGCCACGTTTCTATTACTGACCTACGTGGATCTGGCGCACTACGCCAACTATCTGATACTATTATAGCCCGTGAGCGTAATCAGCAAGGCGATATGCCTAACCTTGTCCTCGTTAGTATTCTCAAGTGCCGCTTTACTGGTGATACTGGTATCGCTGGCTACATGGAATTCAACAAGGAAACCGGATGGCTTGAACCATCAAGTTACTCAGGGGAAGAAGAGTCACACTCAGAGTCAACAGAC                                                                                                                                                                                                                                                                                                       <  1:327713/278‑1`  
    `agatgtgtataagagacatCTTAAGAACCCAGACAAAGGTAAAGCACATGAGGAAGGTCGCCCCGTTTCTATTACTGACCTACGTGGTTCTGGCGCACTACGCCAACTATCTGATACTATTATTGCCCTTGAGCGTAATCAGCAAGGCGATATGCCTAACCTTGTCCTCGTTCGTATTCTCAAGTGCCGCTTTACTGGTGATACTGGTATCGCTGGCTACATGGAATACAACAAGGAAACCGGATGGCTTGAACCATCAAGTTACTCAGGGGAAGAAGAGTCACACTCAGAGTCAACAGAC                                                                                                                                                                                                                                                                                                       <  1:277923/282‑1`  
    `acagggCGTAATTTGTTACCTTAAGAACCCAGACAAAGTGAAAGCACATGAAGAAGGTCGCCCCTTTTCTATTACTGACCTACGTGGTTCTGGCGCACTACGCCAACTATCTGATACTATTATTGCCCTTGCGCGTAATCAGCAAGGCGATATGCCTAACCTTGTCCTCGTTCGTATTCTCAAGTGCCGCTTTACTGGTGATACTGGTATCGCTGGCTACATGGAATACAACAAGGAAACCGGATGGCTTGAACCATCAAGTTACTCAGGGGAAGAAGAGTCACACTCAGAGTCAACAGAC                                                                                                                                                                                                                                                                                                       <  1:465762/295‑1`  
    `aatgtgaagaaaagacagCCTTAAGAACCAAGACAAAGGTAAAGCACAGGAGGAAGGTCGCCCCTTTTCTATTACTGACCTACGTGGTTCTGGCACACTACGCAAACTATCTGATACTATTATTGCCCTTGAGCGTAATCAGCAAGGCGATATGCATAACCTTGTCCTCGTTCGTATTCTCAAGTGCCGCTTTACTGGTGATACTGGTATCGATGGCTACATGGAATACAACAAGGAAACCGGATGGCTTGAACCATCAAGTTACTCAGGGGAAGAAGAGTCACACTCAGAGTCAACAGAC                                                                                                                                                                                                                                                                                                       <  1:318754/283‑1`  
    `GCTGGTCGTAATTTGTCACCTTAAGAACCCATACAAAGGTAAAGCACATGAGGAAGGTCGCCCCGTTTCTATTACTGACCTACGTGGTTCTGGCGCACTACGCCAACTATCTGATACTATTATTGCCCTTGAGCGTAATCAGCAAGGCGATATGCCTAACCTTGTCCTCGTTCGTATTCTCAAGTGCCGCTTTACTGGTGATACTGGTATCGCTGGCTACATGGAATACAACAAGGAAACCGGATGGCTTGAACCATCAAGTTACTCAGGGGAAGAAGAGTCACACTCAGAGGCAACAGAC                                                                                                                                                                                                                                                                                                       <  1:274349/301‑1`  
    `GCTGGTCGTAATTTGTCACCTTAAGAACCCAGACAAAGGTAAAGCACATGAGGAAGGTCGCCCCGTTTCTATTACTGACCTACGTGGTTCTGGCGCACTACGCCAACTATCTGATACTATTATTGCCCTTGAGCGTAATCAGCAAGGCGATATGCCTAACCTTGTCCTCGTTCGTATTCTCAAGTGCCGCTTTACTGGTGATTCTGGTATCGCTGGCTACATGGAATACAACAAGGAAACCGGATGGCTTGAACCATCAAGTTACTCAGGGGAAGAAGAGTCACACTCAGAGTCAACAGAC                                                                                                                                                                                                                                                                                                       <  1:204640/301‑1`  
    `GCTGGTCGTAATTTGTCACCTTAAGAACCCAGACAAAGGTAAAGCACATGAGGAAGGTCGCCCCGTTTCTATTACTGACCTACGTGGTTCTGGCGCACTACGCCAACTATCTGATACTATTATTGCCCTTGAGCGTAATCAGCAAGGCGATATGCCTAACCTTGTCCTCGTTCGTATTCTCAAGTGCCGCTTTACTGGTGATACTGGTATCGCTGGCTACATGGAATACAACAAGGAAACCGGATGGCTTGAACCATCAAGTTACTCAGGGGAAGAAGAGTCACACTCAGAGTCAACAGAC                                                                                                                                                                                                                                                                                                       <  1:23071/301‑1`  
    `GCTGGTCGTAATTTGTCACCTTAAGAACCCAGACAAAGGTAAAGCACATGAGGAAGGTCGCCCCGTTTCTATTACTGACCTACGTGGTTCTGGCGCACCACGCCAACTATCTGATACTATTATTGCCCTTGAGCGTAATCAGCAAGGCGATATGCCTAACCTTGTCCTCGTTCGTATTCTCAAGTGCCGCTTTACTGGTGATACTGGTATCACTGGCTACATGGAATACAACAAGGAAACCGGATGGCTTGAACCATCAAGTTACTCAGGGGAAGAAGAGTCACACTCAGAGTCAACAGAC                                                                                                                                                                                                                                                                                                       <  1:155172/301‑1`  
     `ttgtTCGTAATTTGTCACCTTAAGAACCCAGACAAAGGTAAAGCACATGAGGAAGGTCGCCCCGTTTCTATTACTGACCTACGTGGGTCTGGCGCACTACGCCAACTATCTGATACTATTATTGCCCTTGAGCGTAATCAGCAAGGCGATATGCCTAACCTTGTCCTCGTTCGTATTCTCAAGTGCCGCTTTACTGGTGATACTGGTATCGCTGGCTACATGGAATACAACAAGGAAACCGGATGGCTTGAACCATCAAGTTACTCAGGGGAAGAAGAGTCACACTCAGAGTCAACAGACT                                                                                                                                                                                                                                                                                                      <  1:255522/297‑1`  
     `tgtgtataagagacagACCTTAAGAACCCAGACAAAGGTAAAGCACATGAGGAAGGTCGCCCCGTTTCTATTACTGACCTACGTGGTTCTGGCGCACTACGCCAACTATCTGATACTATTATTGCCCTTGAGCGTAATCAGCAAGGCGATATGCCTAACCTTGTCCTCGTTCGTATTCTCGAGTGCCGCTTTACTGGTGATACTGGTATCGCTGGCTACATGGAATACAACAAGGAAACCGGATGGCTTGAACCATCAAGTTACTCAGGGGAAGAAGAGTCACACTCAGAGTCAACAGACT                                                                                                                                                                                                                                                                                                      <  1:442947/285‑1`  
     `cttgttGTAATTTGGCACCTGAAGAACCCAGACAAATGTAAAGCACATGAGGAAGGTCGCCCCGTTTCTATTACTGCCCTACGTGGTTCTGGCGCACTACTCCAACTATCTGATACTATTCTTGCCCTTGAGCGTAATCAGCAAGGCGATATGCCTAACCTTGTCCTCGTTCGTATTCTCAAGTGCCGCTTTACTGGTGATACTGGTATCGCTGGCTACATGGAATACAACAAGGAAACCGGATGGCTTGAACCATCAAGTTACTCAGGGGAAGAAGAGTCACACTCAGAGTCAACAGACT                                                                                                                                                                                                                                                                                                      <  1:383024/295‑1`  
     `atgtgtataagagacagCCTTAAGAACCCAGACAAAGGTAAAGCACATGAGGAAGGTCGCCCCGTTTCTATTACTGACCTACGTGGTTCTGGCGCACGACGCCAACTATCTGATACGATTATTGCCCTTGAGCGTAATCAGCAAGGCGATATGCCTAACCTTGTCCTCGTTCGTATTCTCAAGTGCCGCTTTACTGGTGATACTGGTATCGATGGCTACATGGAATACAACAAGGAAACAGGATGGCTTGAACCATCAAGTTACTCAGGGGAAGAAGAGTCACACTCAGAGTCAACAGACT                                                                                                                                                                                                                                                                                                      <  1:477663/284‑1`  
     `aaaaagtttataagagacagTAAGAACCCAGACAAATGTAAAGCACATGAGGAAGGTCACCCCGTTTCTATTACTGACCTACGTGGTTCTTGCGCACTACGCCAACTATTTGATACTATTATTGCCCTTGAGCGTAATCAGCAAGGCGATAGGCCTAACCTTTTCCTCGTTCGTATTCTCAAGTGCCGCTTTACTGGTGATACTGGTATCGCTGGCTACATGGAATACAACAAGGAAACCGGATGGCTTGAACCATCAAGTTACTCAGGGGAAGAAGAGTCACACTCAGAGTCAACAGACT                                                                                                                                                                                                                                                                                                      <  1:175099/281‑1`  
     `CTGGTCGTAATTTGTCACCTTAAGAACCCAGACAAAGGTAAAGCACATGAGGAAGGTCTCCCCGTTTCTATTACTGACCTACGTGGTTCTGGCGCACTACGCCAACTATCTGATACTATTATTGCCCTTGAGCGTAATCAGCAAGGCGATATGCCTAACCTTGTCCTCGTTCGTATTCTCAAGTGCCGCTTTACTGGTGATACTGGTATCGCTGGCTACATGGAATACAACAAGGAAACCGGATGGCTTGAACCATCAAGTTACTCAGGGGAAGAAGAGTCACACTCAGAGTCAACAGACT                                                                                                                                                                                                                                                                                                      <  1:115873/301‑1`  
      `TGGTCGTAATTTGTCACCTTAAGAACCCAGACAAAGGTAAAGCACATGAGGAAGGTCGCCCCGTTTATATTACTGACCTACGTGGTTCTGGCGCACTACGCCAACTATCTGATACTATTATTGCCCTTGAGCGTAATCAGCAAGGCGATATGCCTAACCTTGTCCTCGTTCGTATACTCAAGTGCCGCTTTACTGGTGATACTGGTATCGCTGGCTACATGGAATACAACAAGGAAACCGGCTGGCTTGAACCATCAAGTTACTCAGGGGCAGAAGAGTCACACTAAGAGTCAACAGACTG                                                                                                                                                                                                                                                                                                     >  1:88569/1‑301`  
       `gtTCGTAATTTGTCACCTTAAGAACCCAGACAAAGGTAAAGCACATGAGGAAGGTCGCCCCGTTTCTATTACTGACCTACGTGGTTCTGGCGCACTACGCCAACTATCTGATACTATTATTGCCCTTGAGCGTAATCAGCAAGGCGATATGCCTAACCTTGTCCTCGTTCGTATTCTCAAGTGCCGCTTTACTGGTGATACTGGTATCGCTGGCTACATGGAATACAACAAGGAAACCGGATGGCTTGAACCATCAAGTTACTCAGGGGAAGAAGGGTCACACTCAGAGTCAACAGACTGG                                                                                                                                                                                                                                                                                                    <  1:7975/299‑1`  
       `GGTTGTAAGTTGTCACCTTAAGAACCCAGACAAAGGTAAAGTCCATGAGGAAGGTCGCCCCGTTTCTATTACTGACCTACGTTGTTCTGGCGCACTACGCCAACTATCTGATACTATTATTGCCCTTGAGCGTAATCAGCAAGGCGATATGCCTAACCTTGTCCTCGTTCGTATTCTCAAGTGCCTCTTTACTGGTGATACTGGTATCGCTGGCTACATGGAATACAACAAGGAAACCGGATGGCTTGAACCATCAAGTTACTCAGGGGAAGAAGAGTCACACTCAGAGTCAACAGACTGG                                                                                                                                                                                                                                                                                                    <  1:335997/301‑1`  
       `GGTCGTAATTTGTCACCTTAAGAACCCAGACAAATGTAAAGCACATGAGGAAGGTCGCCCCGTTTCTATTACTGACCTACGTGGTTCTGGCGCACTACGCCAACTATCTGATACTATTATTGCCCTTGAGCGTAATCAGCAAGGCGATATGCCTAACCTTGTCCTCGTTCGTATTCTCAAGTGCCGCTTTACTGGTGATACTTGTATCGCTGGCTACATGGAATACAACAAGGAAACCGGATGGCTTGAACCATCAAGTTACTCAGGGGCAGAAGAGTCACACTCAGAGTCAACAGACTGG                                                                                                                                                                                                                                                                                                    >  1:414899/1‑301`  
       `GGTCGTAATTTGTCACCTTAAGAACCCAGACAAAGGTAAAGCACCTGAGGAAGGTCGCCCCGTTTCTATTACTGACCTACGTGGTTCTGGCGCACTACGCCAACTATCTGATACTATTATTGCCCTTGAGCGTAATCAGCAAGGCGATATGCCTAACCTTGTCCTCGTTCGTATTCTCAAGTGCCGCTTTACTGGTGATACTGGTATCGCTGGCTACATGGAATACAACAAGGAAACCGGATGGCTTGAACCATCAAGTTACTCAGGGGAAGAAGAGTCACACTCAGAGTCAACAGACTGG                                                                                                                                                                                                                                                                                                    >  1:358156/1‑301`  
       `GGTCGTAATTTGTCACCTTAAGAACCCAGACAAAGGTAAAGCACATGAGGAAGGTCGCCCCGTTTCTATTACTGACCTACGTGGTTCTGGCGCACTACGCCAACTATCTGATACTATTTTTGCCCTTGAGCGTAATCAGCAAGGCGATATGCCTAACCTTGTCCTCGTTCGTATTCTCAAGTGCCGCCTTACTGGTGATACTGGTATCGCTGGCTACATGGAATACAACAAGGAAACCGGATGGCTTGAACCATCAAGTTACTCAGGGGAAGAAGAGTCACCCTAATAGTCAACAGACTGG                                                                                                                                                                                                                                                                                                    >  1:221407/1‑301`  
       `GGTCGTAATTTGTCACCTTAAGAACCCAGACAAAGGTAAAGCACATGAGGAAGGTCGCCCCGTTTCTATTACTGACCTACGTGGTTCTGGCGCACTACGCCAACTATCTGATACTATTATTGCCCTTGATCGTAATCAGCAAGGCGATATGCCTAACCTTGTCCTCGTTCGTATTCTCAAGTGCCGCTTTACTGGTGATACTGGTATCGCTGGCTACATGGAATACAACAAGGAAACCGGATGGCTTGAACCATCAAGTTACTCAGGGGCAGAAGAGTCACACTCAGAGTCAACAGACTGG                                                                                                                                                                                                                                                                                                    >  1:277639/1‑301`  
       `GGTCGTAATTTGTCACCTTAAGAACCCAGACAAAGGTAAAGCACATGAGGAAGGTCGCCCCGTTTCTATTACTGACCTACGTGGTTCTGGCGCACTACGCCAACTATCTGATACTATTATTGCCCTTGAGCGTAATCAGCAAGGCGATATGCCTAACCTTGTCCTCGTTCGTATTCTCAAGTGCCGCTTTACTGGTGATACTGGTATCGCTGGCTACATGGAATACAACAAGGAAACCGGATGGCTTGAACCATCAAGTTACTCAGGGGAAGAAGAGTCACACTCAGAGTCACCAGACTct                                                                                                                                                                                                                                                                                                    >  1:184799/1‑299`  
       `GGTCGTAATTTGTCACCTTAAGAACCCAGACAAAGGTAAAGCACATGAGGAAGGTCGCCCCGTTTCTATTACTGACCTACGTGGTTCTGGCGCACTACGCCAACTATCTGATACTATTATTGCCCTTGAGCGTAATCAGCAAGGCGATATGCCTAACCTTGTCCTCGTTCGTATTCTCAAGTGCCGCTTTACTGGTGATACTGGTATCGCTGGCTACATGGAATACAACAAGGAAACCGGATGGCTTGAACCATCAAGTTACTCAGGGGAAGAAGAGTCACACCCAGAGTCAACCGACTGG                                                                                                                                                                                                                                                                                                    >  1:8373/1‑301`  
       `GGTCGTAATTTGTCACCTTAAGAACCCAGACAAAGGTAAAGCACATGAGGAAGGTCGCCCCGTTTCTATTACTGACCTACGTGGTTCTGGCGCACTACGCCAACTATCTGATACTATTATTGCCCTTGAGCGTAATCAGCAAGGCGATATGCCTAACCTTGTCCTCGTTCGTATTCTCAAGTGCCGCTTTACTGGTGATACTGGTATCGCTGGCTACATGGAATACAACAAGGAAACCGGATGGCTTGAACCAACCAGTTACTCAGGGGAAGAAGAGTCACACTCAGAGTCAACActgacc                                                                                                                                                                                                                                                                                                    >  1:314574/1‑295`  
       `GGTCGTAATTTGTCACCTTAAGAACCCAGACAAAGGTAAAGCACATGAGGAAGGTCGCCCCGTTTCTATTACTGACCTACGTGGTTCTGGCGCACTACGCCAACTATCTGATACTATTATTGCCCTTGAGCGTAATCAGCAAGGCGATATGCCTAACCTTGTCCTCGTTCGTATTCTCAAGTGCCGCTTTACTGGTGATACTGGTATCGCTAGCTACATGGAATACAACAAGGAAACCGGATGGCTTGAACCATCAAGTTACTCAGGGGAAGAAGAGTCACACTCAGAGTCAACAGACGGG                                                                                                                                                                                                                                                                                                    >  1:33925/1‑301`  
       `GGTCGTAATTTGTCACCTTAAGAACCCAGACAAAGGTAAAGCACATGAGGAAGGTCGCCCCGTTTCTATTACTGACCTACGTGGTTCTGGCGCACTACGCCAACTATCTGATACTATTATTGCCCTTGAGCGTAATCAGCAAGGCGATATGCCTAACCTTGTCCTCGTTCGTATTCTCAAGTGCCGCTTAACTGGTGATACTGGTATCGCTGGCTACATGGAATACAACAAGGAAACCGGATGGCTTGAACCATCAAGTTACTCAGGGGAAGAAGAGTCACACTCAGAGTCAACAGACTGG                                                                                                                                                                                                                                                                                                    >  1:5090/1‑301`  
       `GGTCATTATTTGTCCACTTAAGAACCCAGACAAAGGTAAAGCACATGAGGAAGGTCGACCCGTTTCTATTAATGACCTACGTGGTTCTGGCGCACGACGCCAAATATCTGATACTATTAGTGCCCTTGAGAGTAATCAGCAAGGCGATATGCCTAACCTTGTCCTCGTTCGTATTCTCAAGTGCCGCTTTACTGGTGATACTGGTATCGCTGGCTACATGGAATACAACAAGGAAACCGGATGGCTTGAACCATCAAGTTACTCAGGGGAAGAAGAGTCACACTCAGAGTAAACAGACTGG                                                                                                                                                                                                                                                                                                    <  1:402389/301‑1`  
            `TAATTTGTCACCTTAAGAACCCAGACAAAGGTAAAGCACATGAGGAAGGTCGCCCCGTTTCTATTACTGACCTACGTGGTTCTGGCGCACTACGCCAACTATCTGATACTATTATTGCCCTTGAGCGTAATCAGCAAGGCGATATGCCTAACCTTGTCCTCGTTCGTATTCTCAAGTGCCGCTTTACTGGTGATACTGGTATCGCTGGCTACATGGAATACAACAAGGAAACCGGATGGCTTGAACCATCAAGTTACTCAGGGGAAGAAGAGACACACTCAGAGTCAACAGACctgtctct                                                                                                                                                                                                                                                                                               >  1:153561/1‑293`  
              `ATTTGTCACCTTAAGAACCCAGACAAAGGTAAAGCACATGAGTAAGGTCGCCCCGTTTCTATTACTGACCTACGTGGTTCTGGCGCACTACGCCAACTATCTGAAACTATTATTGCCCTTGAGCGTAATCAGCAAGGCGATATGCCTAACCTTGTCCTCGTTCGTATTCTCAAGTGCCGCTTTACTGGTGATACTGGTATCGCTGGCTACATGGAATACAACAAGGAAACCGGATGGCTTGAACCATCAAGTTACTCAGGGGAAGAAGAGTCACACTCAGAGTCAACAGACTGGCTAGAAG                                                                                                                                                                                                                                                                                             <  1:261855/301‑1`  
              `ATTTGTCACCTTAAGAACCCAGACAAAGGTAAAGCACATGAGGAAGGTCGCCCCGTTTCTATTACTGCCCTACGTGGTTCTGGCGCACTACGCCAACTATCTGATACTATTATTGCCCTTGAGCGTAATCAGCAAGTCGATATGCCTAACCTTATCCTCGTTCGTATTCTCAAGTGCCGCTTTACTGGTGATTCTGGTATCGCTGGCTACATGGAATACAACAAGGAAACCGGATTGCTTGACCCATCAAGTTACTCAGGGGAAGAAGATTCACACTCCGAGTCAACAGACctgtcccttt                                                                                                                                                                                                                                                                                             >  1:39821/1‑291`  
               `TTTGTCACCTTAAGAACCCAGACAAAGGTAAAGCACATGAGGAAGGTCGCCCCGTTTCTATTACTGACCTACGTGGTTCTGGCGCACTACGCCAACTATCTGATACTATTATTGCCCTTGAGCGTAATCAGCAAGGCGATATGACTAACCTTGTCCTCGTTCGTATTCTCAAGTGCCGCTTTACTGGTGATACTGGTATCGCTGGCTCCATGGAATACAACAAAGAAACCGGATGGCTTGAACCATCAAGTTACTCAGGGGAAGAAGAGTCACACTCAGAGTCAACAGAATGGCTGGAAGA                                                                                                                                                                                                                                                                                            >  1:78191/1‑301`  
                `TTGTCACCTTAAGAACCCAGACAAAGGTAAAGCACATGAGGAAGGTCGCCCCGTTTCTATTACTGACCTACGTGGTTCTGGCGCACTACGCCAACTATCTGATACTATTATTGCCCTTGAGCGTAATCAGCAAGGCGATATGCCTAACCTTGTCCTCGTTCGTATTCTCAAGTGCCGCTTTACTGGTGATACTGGTATCGCTGGCTACATGGAATACAACAAGGAAACCGGATGGCTTGAATCATCAAGTTACTCAGGGGAAGAAGAGTCACACTCAGAGTCAACAGACTctgtctcttat                                                                                                                                                                                                                                                                                           >  1:193781/1‑290`  
                      `CCTTAAGAACCCAGACAAAGGTAAAGCACATGAGGAAGGTCTCCCAGTTTCTATTACTGACCTACGTGGTTCTGGCGCACTACGCCAACTATCTGATACTATTATTGCCCTTGAGCGTAATCAGCAAGGCGATATGCCTAACCTTGTCCTCGTTCGTATTCTCAAGTGCCGCTTTACTGGTGATACTGGTATCGCTGGCTACATGGAATACAACAAGGAAACCGGATGGCTTGAACCATCAAGTTACTCAGGGGAAGAAGAGTCACACTCAGAGTAAACctgtctcttatacacatctccg                                                                                                                                                                                                                                                                                     >  1:198106/1‑279`  
                      `CCTTAAGAACCCAGACAAAGGTAAAGCACATGAGGAAGGTCGCCCCGTTTCTATTACTGACCTACGTGGTTCTGGCGCACTACGCCAACTCTCTGATACTATTATTTCCCTTGAGCGTAATCAGCAAGGCGATATGCCTAACCTTGTCCTCGTTCGTATTCTCAAGTGCCGCTTTACTGGTGATACTGGTATCGCTGGCTACATGGAATACAACAAGGAAACCGGATGGCTTGAACCATCAAGTTACTCAGGGGAAGAAGAGTCACACTCAGAGTCAACAGACTctgtctcttatacacat                                                                                                                                                                                                                                                                                     >  1:303965/1‑284`  
                      `CCTTAAGAACCCAGACAAAGGTAAAGCACATGAGGAAGGTCGCCCCGTTTCTATTACTGACCTACGTGGTTCTGGCGCACTACGCCAACTATCTGATCCTATTATTGCCCTTGAGCGTAATCAGCAATGCGATATGCCTAACCTTGTCCTCGTTCGTATTCTCAAGTGCCGCTTTACTGGTGATACTGGTATCGCTGGCTACATGGCATACAACAAGGAAACCGGCTGGCTTGAACCATCAATTTACTCAGGTGAAGAAGCGTCCCCCTCAGAGTCAACAGCCTctgtctcttatccacat                                                                                                                                                                                                                                                                                     >  1:92433/1‑284`  
                       `CTTAAGAACCCAGACAAAGGTAAAGCACATGAGTACGGTCGCCCCGTTTCTATTACTGACCTACGTGGTTCTGGCGCACTACGCCAACTATCTGAACCTATTATAGCCCTTGAGCGTAATCAGCAACGCGATCTGCCTAACCTTTTCCTAGCTCGTATTCTCAAGTGCCGCTTTACTGGTGATACTGGTATCGCTGGCTACATGGAATCCATCAAGGAAACCGGATGGCGTGAACCATCAAGATACTCAGGGGAAGATACGTCCCAATCAGAGTCAACAGACTGctcacacgcacctgtcc                                                                                                                                                                                                                                                                                    >  1:158957/1‑284`  
                         `TAAGAACCCAGACAAAGGTAAAGCACATGAGGAAGGTCGCCCCGTTTCTATTACTGACCTACGTGGTTCTGGCGCACTACGCCAACTATCTGATACTATTATTGCCCTTGAGCGTAATCAGCAAGGCGATATGCCTAACCTTGTCCTCGTTCGTATTCTCAAGTGCCGCTTTACTGGTGATACTGGTATCGCTGGCTTCTTGGAATACAACAAGGAAACCGGATGGATTGAACCATCAAGTTACTCAGGGGAAGAAGAGTCACACTCAGAGTCAACAGACTctgtttcttatacacatctc                                                                                                                                                                                                                                                                                  >  1:492461/1‑281`  
                              `ACCCAGACAAAGGTAAAGCACATGAGGAAGGTCGCCCCGTTTCTATTACTGACCTACGTGGTTCTGGCGCACTACGCCAACTATCTGATACTATTATTGCCCTTGAGCGTAATCAGCAAGGCGATATGCCTAACCTTGTCCTCGTTCGTCTTCTCACGTGCCGCTTTACTGGTTATACTGGTATCGCTGGCTACATGGAATACAACAAGGAAACCGGATGGCTTGAACCATCAAGTTACTCAGTGTAAGAAGAGTCACACTCATAGTCAACctgtctcttaaccacatctccgagcacacc                                                                                                                                                                                                                                                                             >  1:256671/1‑271`  
                               `CCCAGACAAAGGTAAAGCACATGAGGAAGGTCGCCCCGTTTCTATTACTGACCTACGTGGTTCTGGCGCACTACGACAACTATATGATACTATTATTGCCCTTGAGAGTAATCAGCAAGGCGATATGCCTAACCTTGTCCTCGTTCGTATTCTCAAGTGCCGCTTTACTGGTGATACTGGTATGCCTGGGTACTTGGAATACAACAAGGAAACCGGATGGGTTGAACCATCAGGATACCGAGGGGAGGAAGATTCATACTCAGAGTCAACAGcatggtccaagaacacggccctcggacag                                                                                                                                                                                                                                                                            >  1:440856/1‑272`  
                                        `AGGTAAAGCACATGAGGAAGGTCGCCCCGTTTCTATTACTGACCTACGTGGTTCTGGCGCACTACGCCAACTATCTGATACTATTATTGCCCTTGAGCGTAATCAGCAAGGCGATATGCCTAACCTTGTCCTCGTTCGTATTCTCAAGTGCCGCTTTACTGGTGATACTGGTATCGCTGGCTACATGGAATACAACAAGGAAACCGGATGGCTTGAACCATCAAGTTACTCAGGGGAAGAAGAGTCACACTCAGAGTCAACAGACTGGCTGGAAGAAACCctgtctcttatacacatctcc                                                                                                                                                                                                                                                                   >  1:386047/1‑280`  
                                             `AAGCACATGAGGAAGGTCGCACCGTTTCTATTACTGACCGACGTGGTTCTGGCGAACTACGCAAACTATCTGATACTATTATTTCCCTTGAGCGTAATCAGCAAGGCGATATGCCTAACCTTGTCCTCGTTCGTATTCTCAAGGGCCGCTTTACTGGTGAGACTGGTATCGCTGGCTACATGGAATACAACAAGGAAACCGGATGGCTTGAACCATCAAGTTACTCAGGTGAAGAAGAGTCACACTCAGAGTCAACAGACTGGCTGGAAGAAACCAGAAGTTACCAAGCTGAGTCATAAGG                                                                                                                                                                                                                                                              <  1:145166/301‑1`  
                                                               `aagagacagCTATTTCTGACCTACGTGGTTCTGGCGCACTACGCCAACTATCTGATACTATTAGTGCCCTTGAGCGTAATCAGCAAGGCGATATGCCTAACCTTGTCCTCGTTCGTATTCTCAAGTGCCGCTTTACTGGTGATACTGGTATCGCTGGCTACATGGAATACAACAAGGAAACCGGATTGCTTGAACCATCAAGTTACTCAGGGGAAGAAGAGTCACACTCAGAGTCAACAGACTGGCTGGAAGAAACCAGAAGTTACCAAGCTGAGTCATAAGGTGTTCGATAAGGACACTA                                                                                                                                                                                                                                            <  1:194603/292‑1`  
                                                               `aagagacagCTATTACTGACCTACGTGGTTCTGGCGCACTACGCCAACTATCTGATACTATTATTGCCCTTGAGCGTAATCAGCAAGGCGATATGCCTAACCTTGTCCTCGTTCGTATTCTCAAGTGCCGCTTTACTGGTGATACTGGTATCGCTGGCTACATGGAATACAACAAGGAAACCGGATTGCTTGAACCATCAAGTTACTCAGGGGAAGAAGAGTCACACTCAGAGTCAACAGACTGGCTGGAAGAAACCAGAAGTTACCAAGCTGAGTCATAAGGTGTTCGATAAGGACACTA                                                                                                                                                                                                                                            <  1:194612/292‑1`  
                                                                    `GTTACTATTACTGACCTACGTGGTTCTGGCGCACTACGCCAACTATCTGATACTATTATTGCCCTTGAGCGTAATCAGCAAGGCGATATGCCTAACCTTGTCCTCGTTCGTATTATCAAGTGCCGCTTTACTGGTGATACTGGTATAGCTGGCTACATGGAATACAACAAGGAAACCGGATGGCTTTAACCATCAAGTTACTCAGGGGAAGAAGAGTCACACTCAGATTCAACAGACTGGCTGGACGAAACCAAAAGTTACCAAGCTGAGTCATAAGGTGTTCGATAAGGCACCTATGACC                                                                                                                                                                                                                                       >  1:74205/1‑301`  
                                                                     `TTTCTATTACTGACCTACGTGGTTCTGGCGCACTACGCCAACTATCTTATACTATTATTGCCCTTGAGCGTAATCATCAAGGCGATATTCCTAACCTTGTCCTCGTTCGTATTCTCACGTGCCGCTTTACTGTTGCTCCTGGTCTCGCTGGCTACATTGCCTACAACACGTACACCGGATGTCTTTACCCATCAAGTTACTCAGGGGAAGAAGAGTCACACTCAGCGTCAACAGACTGGCTGGAAGAAACCAGAAGTTACCAAGCTGAGTAATAAGGTATTCGGTCAGGACACTATGACCC                                                                                                                                                                                                                                      >  1:230464/1‑301`  
                                                                      `TTCTATTACTGACCTACGTGGTTCTGGCGCACTACGCCAACTATCTGATACTATTATTGCCCTTGAGCGTAATCAGCAAGGCGATATGCCTAACCTTGTCCTCGTTCGTATTCTCAAGTGCCGCTTTACTGGTGATACTGGTATCGCTGGCTACATGGAATACAACAAGGAAACCGGATGGCTTGAACCATCAAGTTACTCAGGGGAAGAAGAGTCACACTCAGAGTCAACCGACTGGCTGGAAGAAACCAGAAGTTACCAAGCTGAGTCATAAGGTGTTCGATAAGGACACTATGACCCc                                                                                                                                                                                                                                     >  1:373141/1‑300`  
                                                                           `TTACTGACCTACGTGGTTCTGGCGCACTACGCCAACTATCTGATACTATTATTGCCCTTGAGCGTAATCAGCAAGGCGATATGCCTAACCTTGTCCTCGTTCGTATTCTCAAGTGCCGCTTTACTGGTAATACTGGTATCGCTGGCTACATGGAATACAACAAGGAAACCGGATGGCTTGAACCATCAAGTTACTCAGGGAAAGAAGAGTCACACTCAGAGTCAACAGACTGGCTGGAAGAAACCAGAAGTTACCAAGCTGAGTCATAAGGTGTTCGATctgtctcttatacacatctcct                                                                                                                                                                                                                                >  1:214399/1‑279`  
                                                                                      `agagggTCTGGCGAACTACGCCAACTATGTGATACTATTATTGCCCTTGAGCGTAATCAGCAAGGCGATATGCCTAACCTTGTCCTCATTCGTATTCTCAAGTGCCGCTTTACTGGTGATACTGGTATCGCTGGCTACATGGAATACAACAAGGAAGCCAGATGGCTTGAACCATCAAGTTACTCAGGGGAAGAAGAATCACACTCAGAGTCAACAGACTGGCTGGAAGAAACCAGAAGTTACCAAGCTGAGTCATAAGGTGTTCGATAAGGACACTATGACCCACATCAAGGCTGGTGAT                                                                                                                                                                                                                     <  1:408442/295‑1`  
                                                                                          `GTTCTGGCGCACTACGCCAACTATCTGATACTATTATTGCCCTTGAGCGTAATCAGCAAGGCGATATGCCTAACCTTGTCCTCGTTCGTATTCTCAAGTGCCGCTTTACTGGTGATACTGGTATCGCTGGCTACATGGAATACAACAAGGAAACCGGATGGCTTGAACCATCAAGTTACTCAGGGGAAGAAGAGTCACACTCAGAGTCAACAGACTGGCTGGAAGAAACCAGAAGTTACCAAGCTGAGTCATAAGGTGTTAGATAAGGACACTATGACCCACATCAAGGCTGGTGATTGGG                                                                                                                                                                                                                 >  1:380559/1‑301`  
                                                                                          `GTTCTGGCGCACTACGCCAACTATCTGATACTATTATTGCCCTTGAGCGTAATCAGCAAGGCGATATGCCTAACCTTGTCCTCGTTCGTATTCTCAAGTGCCGCTTTACTGGTGATACTGGTATCGCTGGCTACATGGAATACAACAAGGAAACCGGATGGCTTGAACCATCAAGTTACTCAGGGGAAGAAGAGTCACACTCAGAGTCAACAGACTGGCTGGAAGAAACCAGAAGTTACCAAGCTGAGTCATAAGGGGTTCGATAAGGACCCTATGACCCACATCAAGGCTGGTGATTGGG                                                                                                                                                                                                                 >  1:300814/1‑301`  
                                                                                                              `CTATCTGATACTATTATTGCCCTTGAGCGTAATCAGCAAGGCGATATGCCTAACCTTGTCCTCGTTCGTATTCTCAAGTGCCGCTTTACTGGTGATACTGGTATCGCTGGCTACATGGAATACAACAATGAAACCGGATGGCTTGAACCATCAAGTTACTCAGGGGAAGAAGAGTCACACTCAGAGTCAACAGACTGGCTGGAAGAAACCAGAAGTTACCAAGCTGAGTCATAAGGTGTTCGATAAGGACACTATGACCCACATCAAGGCTGGTGATTGGGTTctgtctcttatacacatc                                                                                                                                                                                             >  1:222556/1‑283`  
                                                                                                              `CTATCTGATACTATTATTGCCCTTGAGCGTAATCAGCAAGGCGATATGCCTAACCTTGTCCTCGTTCGTATTCTCAAGTGCCGCTTTACTGGTGATACTGGTATCGCTGGCTACATGGAATACAACAAGGAAACCGGATGGCTTGAACCATCAAGTTACTCAGGGGAAGAAGAGTCACACTCAGAGTCAACAGACTGGCTGGAAGAAACCAGAAGTTACCAAGCTGAGTCATAAGGTGTTCGATAAGGACACTATGACCCACATCAAGGCTGGTGATTGGGTTAAGGTTGACATGGGAGTg                                                                                                                                                                                             >  1:468646/1‑300`  
                                                                                                                `ATCTGATACTATTATTGCCCTTGAGCGTAATCAGCAAGGCGATATGCCTAACCTTGTCCTCGTTCGTATTGTCAAGTGCCGCTTTACTGGTGATCCTGGTATCGCTGGCTACATGGAATACAACAAGGAAACCGGATGGCTTGAACCATCAAGTTACTCAGGGGAAGAAGAGTCACACTCAGAGTCAACAGACTGGCTGGAAGAAACCAGAAGTTACCAAGCTGAGTCATAAGGGGGTCGATAAGGACACTATGACACACATCAAGGCTGGTGATTGGGTTAAGGTTGACATGGGAGTTGT                                                                                                                                                                                           >  1:129393/1‑301`  
                                                                                                                        `CTATTATTGCCCTTGAGCGTAATCAGCAAGGCGATATGCCTAACCTTGTCCTCGTTCGTATTCTCAAGTGCCGCTTTACTGGTGATACTGGTATCGCTGGCTACATGGAATACAACAAGGAAACCGGATGGCTTGAACCATCAAGTTACTCAGGGGAAGAAGAGTCACACTCAGAGTCAACAGACTGGCTGGAAGAAACCAGAAGTTACCAAGCTGAGTCATAAGGTGTTCGATAAGGACACTATGACCCACATCAAGGCTGGTGATTGGGTctgtctcttatacacatctccgagcccac                                                                                                                                                                                   >  1:200123/1‑272`  
                                                                                                                                 `CCCTTGAGCGTAATCAGCAAGGCGATATGCCTAACCTTGTCCTCGTTCGTATTCTCAAGCGCCGCTTTACTGGTGATACTGGTATCCCTGCCTACATGGAATACAACAAGGAAACCGGATGGCTTGAACCATCAAGTTACTCAGGGGAAGAAGAGTCACACTCAGAGTCAACAGACTGGCTGGTAGAAACCAGAAGCTACCAAGCTGAGTCATAAGGAGTTCGATAAGGACACTATGACCCACATCAAGGCTGGTGATTGGGTTAAGGTTGACATGGGAGTTGTTGGTGGATACGGCTACG                                                                                                                                                                          >  1:467513/1‑301`  
                                                                                                                                   `gctcggaaatgtgtatgagagacaggggCTAACCTTGGCCTCGTGAGTATTCTCAAGTGCCGCGTTACTGGTGATACTGGTATCGCTGTCTACATGGAATACAACAAGGAAACCGGATGGCTTGAACCATCAAGTTACTCAGGGGAAGAAGAGACACACTCAGAGTCAACAGACTGGCTGGAAGAAACCAGAAGTTACCAAGCTGAGTCATAAGGTGTTCGATAAGGACACTATGACCCACATCAAGGCTGGTGATTGGGTTAAGGTTGACATGGGAGTTGTTGGTGGATACGGCTAAGTC                                                                                                                                                                        <  1:438449/273‑1`  
                                                                                                                                   `CTTGAGCGTAATCAGCAAGGCGATATGCCTAACCTTGTCCTCGTTCGTATTCTCAAGTGCCGCTTTACTGGTGATACTGGTATCGCTGGCTACATGGAATACAACAAGGAAACAGGATGGCTTGAACCATCAAGTTACTCAGGGGAAGAAGAGTCACACTCAGAGTCAACAGACTGGCTGGAAGAAACCAGAAGTTACCAAGCTGAGTCATAAGGTGTTCGATAAGGACACTATGACCCACATCAAGGCTGGTGATTGGGTTAAGGTTGACATGGGAGTTGTTGGTGGATACGGCTACGTC                                                                                                                                                                        <  1:179662/301‑1`  
                                                                                                                                                  `CAAGGCGATATGCCTAACCTTGTCCTCGTTCGTATTCTCAAGGGCCGCTTTACGGGTGATACTGGTATCGCTGGCTACATGGAATACAACAAGGAAACCGGATGGCTTGAACCATCAAGTTACTCAGGGGAAGAAGAGTCACACTCAGAGTCAACAGACTGGCGGGAAGAAACCAGAAGTTACCAAGCTGAGTCATAAGGTGTTCGATAAGGACACTATGACCCACATCAAGGCTGGTGATTGGGTTAAGGTTGACATGGGAGTTGTTGGTGGATACGGCTACGTCCGCTCAGTTAGTGGC                                                                                                                                                         <  1:229396/301‑1`  
                                                                                                                                                      `GCGATATGCCTAACCTTGTCCTCGTTCGTATTCTCAAGTGCCGCTTTACTGGTGATACTGGTATCGCTGGCTACATGGAATACAACAAGGAAACCGGATGGCTTGAACCATCAAGTTACTCAGGGGAAGCAGAGTCACACTCAGAGTCAACAGACTGGCTGGAAGAAACCAGAAGTTACCAAGCTGAGTCATAAGGTGTTCGATAAGGACACTATGACCCACATCAAGGCTGGTGCTTGGGTTAAGGTTGACATGGGAGTTGTTGGTGGATACGGCTACGTCCGCTCAGTTAGTGGCActg                                                                                                                                                     >  1:162556/1‑298`  
                                                                                                                                                            `TGCCTAACCTTGTCCTCGTTCGTATTCTCAAGTGCCGCTTTACTGGTGATACTGGTATCGCTGGCTACATGGAATACAAAAAGGAAACCGGATGGCTTGAACCATCAAGTTACTCAGGGGAAGAAGAGTCACACTCAGAGTCAACAGACTGGCTGGAAGAAACCAGAAGTTACCAAGCTGAGTCATAAGGTGTTCGATAAGGACACTATGACCCACATCAAGGCTGGTGATTGGGTTAAGGTTGACATGGGAGTTGTTGGTGGATACGGCTACGTCCGCTCAGTTAGTGGCAAATATGCAC                                                                                                                                               <  1:221718/301‑1`  
                                                                                                                                                             `GCCTAACCTTGTCCTCGTTCGTATTCTCAAGTGCCGCTTTACTGGTGATACTGGTATCGCTGGCTACATGGAATACAACAAGGAAACCGGATGGCTTGAACCATCAAGTTACTCAGGGGAAGAAGAGTCACACTCAGAGTCAACAGACTGGCTGGAAGAAACCAGAAGTTACCAAGCTGAGTCATAAGGTGTTCGATAAGGACACTATGACCCACATCAAGGCTGGTGATTGGGTTAAGGTTGACATGGGAGTTGTTGGTGGATACGGCTACGTCCGCTCAGTTAGTGGCAAATATGCACA                                                                                                                                              >  1:8724/1‑301`  
                                                                                                                                                             `GCCTAACCTTGTCCTCGTTAGTATTCTCAAGTGACGCTTTACTGGTGATACTGGTATCGCTGGCTACATGGAATACAACAAGGAAACCGGATGGCTTGAACCATCAAGTTACTCAGGGGAAGAAGAGTCACACTCAGAGTCAACAGACTGGCTGGAAGAAACCAGAAGTTACCAAGCTGAGTCATAAGGTGTTCGATAAGGACACTATGACCCACAACAAGGCTGGTGATTGGGTTAAGGTTGACATGGGAGTTGTTGGTGGATACGGCTACGTCCGCTCAGTTctgtatcttatacacat                                                                                                                                              >  1:55510/1‑284`  
                                                                                                                                                                         `CCTCGTTCGTATTCGCAAGTGCCGCTTTACTGGTGATACTGGTATCGATGGCTACATGGAATACAACAAGGAAACCGGATGGCTTGAACCATCAAGTTACTCAGGGGAAGAAGAGTCACACTCAGAGTCAACATACTGGCTGGAAGAAACCAGAAGTTACCAAGCTGAGTCATAAGGTGTTCGATAAGGACACTATGACCCACATCAAGGCTGGTGATTGGGTTAAGGTTGACATGGGAGTTGTTGGTGGATACGGCTACGTCCGCTCAGTTAGTGGCAAATATGCACAAGTGTCATACAT                                                                                                                                  <  1:464065/301‑1`  
                                                                                                                                                                            `CGTTCGTATTCTCAAGTGCCGCTTTACTGGTGATACTGGTATCGCTGGCTACATGGAATACAACAAGGAAACCGGATGGCTTGAACCATCAAGTTACTCAGGGGAAGAAGAGTCACACTCAGAGTCAACAGACTGGCTGGAAGAAACCAGAAGTTACCAAGCTGAGTCATAAGGTGTTCGATAAGGACACTATGACCCACATCAAGGCTGGTGATTGGGTTAAGGTTGACATGGGAGTTGTTGGTGGATACGGCTACGTCCGCTCAGTTAGTGGCAAATATGCACAAGTGTCATACATCAC                                                                                                                               >  1:88242/1‑301`  
                                                                                                                                                                                 `GTATTCTCAAGTGCCGCTTTACTGGTGATACTGGTATCGCTGGCTACATGGAATACAACAAGGAAACCGGATGGCTTGAACCATCAAGTTACTCAGGGGAAGAAGAGTCACACTCAGAGTCAACAGACTGGCTGGAAGAAACCAGAAGTTACCAAGCTGAGTCATAAGGTGTTCGATACGGCCACTATGACCCACATCAAGGCTGGTGCTTGGGTTAAGGTTGACATGGGATCTGTTGGTGAATACGGCTACGTCCGATCAGTTAGTGGCAAATATGCACAAGTGTCATACATCtgtctct                                                                                                                          >  1:284506/1‑294`  
                                                                                                                                                                                       `atgtgtataagagacagGGTGATACTGGTATCGCTGGCTACATGGAATACAACAAGGAAACCGGATGGCTTGAACCATCAAGTTACTCAGGGGAAGAAGAGTCACACTCAGAGTCAACAGACTGGCTGGAAGAAGCCAGAAGTTACCAAGCTGAGTCATAAGGTGTTCGATAAGGACACTATGACCCACATCAAGGCTGGTGATTGGGTTAAGGTTGACATGGGAGTTGTTGGTGGATACGGCTACGTCCGCTCAGTTAGTGGCAAATATGCACAAGTGTCATACATCACAGGTGTTACTC                                                                                                                    <  1:450825/284‑1`  
                                                                                                                                                                                                                                  `cgaagagaaccataAAACCGGATTGCTTGAACCATCAAGTTACTCAGTGGAAGAAGAGTCACACTCAGAGTAAACAGAAAAGCTGGAAGAAACCAGAAGTTACCAAGCTGAGTCATAAGGTGTTAGAAAAGGACACTATGACCCACATCAAGGCTGGTGATTGGGTTAAGGTTGACATGGGAGTTGTTGGTGGATACGGCTACGTCCGCTCAGTTAGTGGCAAATATGTACAAGTGTCATACATCACAGGTGTTACGCCACGCGTTGCAATCGTTGCCGATAAGACCAACATGATTCACAC                                                                         <  1:230833/287‑1`  
                                                                                                                                                                                                                                       `ACAATAAGGAAACCGGATGGCTTGAACCATCAAGTTACTCAGGGGAAGAAGAGTCACACTCAGAGTCAACAGACTGGCTGGAAGAAACCAGAAGTTACCAAGCTGAGTCATAAGGTGTTCGATAAGGACACTATGACCCACATCAAGGCTGGTTATTGGGTTAAGGTTGACGTGGGAGTTGTTGGTGGATACGGCTACGTCCGCTCAGTTAGTGGCCAATATGCACAAGTGTCATACATCACAGGTGTTACTCCACGCGTGTCAATCTTTGCCGATAAGACCAACATGATTCACCCAGGTT                                                                    >  1:157411/1‑301`  
                                                                                                                                                                                                                                                         `GGCTTGAACCATCAAGTTACTCAGGGGAAGAAGAGTCACACTCAGAGTCAACAGACTGGCTGGAAGAAACCAGAAGTTACCAAGCTGAGTCATAAGGTGTTCGATAAGGACACTATGACCCACATCAAGGCTGGTGATTGGGTTAAGGTTGACATGGGAGTTGTTGGTGGATACGGCTACGTCCGCTCAGTTAGTGGCAAATATGCACAAGTGTCATACATCACAGGTGTTACTCCACGCGGTGCAATCGTTGCCGATAAGACCAACATGATTCACACAGGTTTCTTGACAGTTGTTTCAT                                                  >  1:477187/1‑301`  
                                                                                                                                                                                                                                                                                 `tGGATGAAGAGTCACACTCAGAGTCAACAGACTGGCTGGAAGAAACCATAAGTTACCAAGCTTAGTCATAAGGTGTTCGATAAGGACACTATGACCCACATCAAGGCTGGTGATTGGGTTAAGGTTGACATGGGAGTTGTTGGTGGATACTGCTACGTCCGCTCAGTTAGTGGCAAATATGCACAAGTGTCATACATCACAGGTGTTACTCCACGCGGTGCAATCGTTGCCGATAAGACCAACATGATTCACACAGGTTTCTTGACAGTTGTTTCATATGAAGAGATTGTTAAGTCACGAT                          <  1:148349/300‑1`  
                                                                                                                                                                                                                                                                                 `GGGAAGAAGAGTCACACTCAGAGTCAAAAGAATGGCTGGAAGAAACCAGAAGTTACCAAGCTGAGTCATAAGGTGTTCGATAAGGACACTATGACCCACATCAAGGCTGGTGATTGGGTTAAGGTTGACATGGGAGTTGTTGGTGGATACTGCTACGTCCGCTCAGTTAGTGGCAAATATGCACAAGTGTCATACATCACAGGTGTTACTCCACGCGGTGCAATCGTTGCCGATAAGACCAACATGATTCACACAGGTTTCTTGACAGTTGTTTCATATGAAGAGATTGTTAAGTCACGAT                          <  1:148370/301‑1`  
                                                                                                                                                                                                                                                                                  `GGAAGAAGAGTCACACTCAGAGTCAACAGACTGGCTGGAAGAAACCAGAAGTTACCAAGCTGAGTCATAAGGTGTTCGATAAGGACACTATGACCCACATCAAGGCTGGTGATTGGGTTAAGGTTGACATGGGAGTTGTTGGTGGATACGGCTACGTCCGCTCAGTTAGTGGCAAATATGCACAAGTGTCATACATCACAGGTGTTACTCCACGCGGTGCAATCGTTGCCGATAAGACCAACATGATTCACACAGGTTTCTTGACAGTTGTTTCATATGAAGAGATTGTTAAGTAACGATA                         <  1:447558/301‑1`  
                                                                                                                                                                                                                                                                                      `GAAGAGTCACACTCAGAGTCAACAGACTGGCTGTAAGAAACCAGAAGTTACCAAGCTGAGTCATAAGGTGTTCGATAAGGACACTATGAACCACATCAAGGATGGTGATTGGGTGAAGGTTGAAATGGGAGTTGTTGGTGGATACGGCTACGTCCGCTCAGTTAGTGGCAAATATGCACAAGTGTCATAAATCGCAGGTGTTAATCCACGCGGTGCAATCGTTGCCGATAAGACCAACATGATTCCAAAAGGTTTCTTGACAGTTGGTTCATATAAAGAGATTGTTAAGTCAAGATAAGCA                     >  1:346770/1‑301`  
                                                                                                                                                                                                                                                                                        `aagacagCACTCAGAGTAAACAGACTGGCTGGAAGAAACCAGAAGTTACCAAGCTGAGTCATAAGGTGTTCGATAAGGACACTATGACCCACATCAAGGCTGGTGATTGGGTTAAGGTTGACATGGGAGTTGTTGGTGGATACGGCTACGTCCGCTCAGTTAGTGGCAAATATGCACAAGTGTCATACATCACAGGTGTTACTCCACGCGGTGCAATCGTTGCCGATAAGACCAACATGATTCACACAGGTTTCTTGACAGTTGTTTCATATGAAGAGATTGTTAAGTCACGATAATCAAT                   <  1:407804/294‑1`  
                                                                                                                                                                                                                                                                                         `GAGTCATACTCAGAGTCAACAGACTGGCTGGAAGAAACCAGAAGTTACCAAGCTGAGTCATAAGGTGTTCGATAAGGACACTATGACCCACATCAAGGCTGGTGATTGGGTTAAGGTTGACATGGAAGTTGTTGGTGGATACGGCTACGTCCGCTCAGTTAGTGGCAAATATGCACAAGTGTCATACATCACAGGTGTTACTCCACGCGGTGCAATCGTTGCCGATAAGACCAACATGATTCACACAGGTTTCTTGACAGTTGTTTCATATGAAGAGATTGTTAAGTCACGATAATCACTA                  >  1:435750/1‑301`  
                                                                                                                                                                                                                                                                                             `CACACTAAGAGTCAACGGACTGGATGGAAGAAACCAAAAGTTACCAAGTTGATTCATAAGGTGTTCGATAAGGACACTATGACCAACATCAAGGCTGGTGATTGGGTTAAGGTTGACATGGGAGTTGTTGTTGGATACGGCTACTCCTGCTCATTTAGTGGCAAATATGCACAAGTGTCATACATCACAGGTGTTACTCCACGCGGTGCAATCGTTGCCGATAAGACCAACATGATTCACACAGGTTTCTTGACAGTTGTTTCATATGAAGAGATTGTTAAGTCACGATAATCAATAGGAG              <  1:302816/301‑1`  
                                                                                                                                                                                                                                                                                                      `AGTCAACAGACTGGCTGGAAGAAACCAGAAGTTACCAAGCTGAGTCATAAGGTGTTCGATAAGGACACTATGACCCACATCAAGGCTGGTGATTGGGTTAAGGTTGACATGGGAGTTGTTGGTGGATACGGCTACGTCCGCTCAGTTAGTGGCAAATATGCACAAGTGTCATACATCACAGGTGTTACTCCACGCGGTGCAATCGTTGCCGATAAGACCAACATGATTCACACAGGTTTCTTGACAGTTGTTTCATATGAAGAGATTGTTAAGTCACGATAATCAATAGGAGctgtctctt     >  1:60962/1‑292`  
                                                                                                                                                                                                                                                                                                         `CAACAGACTGGCTTGAAGAAACCAGAAGTTACCAAGCTGAGTCATAAGGTGTTCGATAAGGACACTATGACCCACATCAAGGCTGGTGATTGGGTTAAGGTTGACATGGGAGTTGTTGGTGGATACGGCTACGTCCGCTCAGTTAGTGGCAAATATGCACAAGTGTCATACATCACAGGTGTTACTCCACGCAGTGCAATCGTTGCCGATAAGACCAACATGATTCACACAGGTTTCTTGACAGTTGTTTCATATGAAGAGATTGTTAAGTCACGATAATCAATctgtctcttatacacat  >  1:24584/1‑284`  

                                                                                                                                                                                                                                                                                                                                                                                                                                                                                                                                                                                                                        
`TGGTGCTGGTCGTAATTTGTCACCTTAAGAACCCAGACAAAGGTAAAGCACATGAGGAAGGTCGCCCCGTTTCTATTACTGACCTACGTGGTTCTGGCGCACTACGCCAACTATCTGATACTATTATTGCCCTTGAGCGTAATCAGCAAGGCGATATGCCTAACCTTGTCCTCGTTCGTATTCTCAAGTGCCGCTTTACTGGTGATACTGGTATCGCTGGCTACATGGAATACAACAAGGAAACCGGATGGCTTGAACCATCAAGTTACTCAGGGGAAGAAGAGTCACACTCAGAGTCAACAGACTGG‑‑‑‑‑‑‑‑‑‑‑‑‑‑‑‑‑‑‑‑‑‑‑‑‑‑‑‑‑‑‑‑‑‑‑‑‑‑‑‑‑‑‑‑‑‑‑‑‑‑‑‑‑‑‑‑‑‑‑‑‑‑‑‑‑‑‑‑‑‑‑‑‑‑‑‑‑‑‑‑‑‑‑‑‑‑‑‑‑‑‑‑‑‑‑‑‑‑‑‑‑‑‑‑‑‑‑‑‑‑‑‑‑‑‑‑‑‑‑‑‑‑‑‑‑‑‑‑‑‑‑‑‑‑‑‑‑‑‑‑‑‑‑‑‑‑‑‑‑‑‑‑‑‑‑‑‑‑‑‑‑‑‑‑‑‑‑‑‑‑‑‑‑‑‑‑‑‑‑‑‑‑‑‑‑‑‑‑‑‑‑‑‑‑‑‑‑‑‑‑‑‑‑‑‑‑‑‑‑‑‑‑‑‑‑‑‑‑‑‑‑‑‑‑‑‑‑‑‑‑‑‑‑‑‑‑‑‑‑‑‑‑‑‑‑‑‑‑‑‑‑‑‑‑‑‑‑‑‑‑‑‑‑‑‑‑‑‑‑‑‑‑‑‑‑‑‑‑‑‑‑‑‑‑‑‑‑‑‑‑  >  NC_001604.1/12937‑13244`  
`‑‑‑‑‑‑‑‑‑‑‑‑‑‑‑‑‑‑‑‑‑‑‑‑‑‑‑‑‑‑‑‑‑‑‑‑‑‑‑‑‑‑‑‑‑‑‑‑‑‑‑‑‑‑‑‑‑‑‑‑‑‑‑‑‑‑‑‑‑‑‑‑‑‑‑‑‑‑‑‑‑‑‑‑‑‑‑‑‑‑‑‑‑‑‑‑‑‑‑‑‑‑‑‑‑‑‑‑‑‑‑‑‑‑‑‑‑‑‑‑‑‑‑‑‑‑‑‑‑‑‑‑‑‑‑‑‑‑‑‑‑‑‑‑‑‑‑‑‑‑‑‑‑‑‑‑‑‑‑‑‑‑‑‑‑‑‑‑‑‑‑‑‑‑‑‑‑‑‑‑‑‑‑‑‑‑‑‑‑‑‑‑‑‑‑‑‑‑‑‑‑‑‑‑‑‑‑‑‑‑‑‑‑‑‑‑‑‑‑‑‑‑‑‑‑‑‑‑‑‑‑‑‑‑‑‑‑‑‑‑‑‑‑‑‑‑‑‑‑‑‑‑‑‑‑‑‑‑‑‑‑‑‑‑‑‑‑‑‑‑‑‑‑‑‑‑‑‑‑‑‑‑‑‑‑‑‑‑‑‑‑‑‑‑‑‑‑‑‑acagactggCTGGAAGAAACCAGAAGTTACCAAGCTGAGTCATAAGGTGTTCGATAAGGACACTATGACCCACATCAAGGCTGGTGATTGGGTTAAGGTTGACATGGGAGTTGTTGGTGGATACGGCTACGTCCGCTCAGTTAGTGGCAAATATGCACAAGTGTCATACATCACAGGTGTTACTCCACGCGGTGCAATCGTTGCCGATAAGACCAACATGATTCACACAGGTTTCTTGACAGTTGTTTCATATGAAGAGATTGTTAAGTCACGATAATCAATAGGAGAAATCAATATGA  >  NC_001604.1/14067‑14356`

  

Alignment Legend

Aligned base mismatch/match (shaded by quality score): `ATCG/ATCG` `< 3 ≤` `ATCG/ATCG` `< 10 ≤` `ATCG/ATCG` `< 19 ≤` `ATCG/ATCG` `< 33 ≤` `ATCG/ATCG` `< 38 ≤` `ATCG/ATCG`

Unaligned base: `atcg`    Masked matching base: `atcg`    Alignment gap: `‑`    Deleted base: `‑`

Reads not counted as support for junction

read\_name Not counted due to insufficient overlap past the breakpoint.

read\_name Not counted due to not crossing MOB target site duplication.
